# Transcriptome analysis of genes associated with autolysis of *Coprinus comatus*

**DOI:** 10.1038/s41598-022-06103-z

**Published:** 2022-02-15

**Authors:** Hong-Bo Guo, Zhi-Fei Zhang, Jia-Qing Wang, Si-Yu Wang, Ji-Kang Yang, Xi-Yao Xing, Xiao-Jian Qi, Xiao-Dan Yu

**Affiliations:** 1 College of Life Engineering, Shenyang Institute of Technology, Fushun, 113122 China; 2 College of Biological Science and Technology, Shenyang Agricultural University, Shenyang, 110866 China

**Keywords:** Genetics, Microbiology

## Abstract

*Coprinus comatus*, widely known as “Jituigu”, is an important commodity and food in China. The yield of *C. comatus*, however, is substantially reduced by the autolysis of the fruiting bodies after harvest. To gain insight into the molecular mechanism underlying this autolysis, we divided the growth of *C. comatus* fruiting bodies into four stages: infant stage (I), mature stage (M), discolored stage (D), and autolysis stage (A). We then subjected these stages to de novo transcriptomic analysis using high-throughput Illumina sequencing. A total of 12,946 unigenes were annotated and analyzed with the Gene Ontology (GO), Clusters of Orthologous Groups of proteins (COG), and Kyoto Encyclopedia of Genes and Genomes (KEGG). We analyzed the differentially expressed genes (DEGs) between stages I and M, M and D, and D and A. Because the changes from M to D are thought to be related to autolysis, we focused on the DEGs between these two stages. We found that the pathways related to metabolic activity began to vary in the transition from M to D, including pathways named as autophagy—yeast, peroxisome, and starch and sucrose metabolism. This study also speculates the possible process of the autolysis of *Coprinus comatus*. In addition, 20 genes of interest were analyzed by quantitative real-time PCR to verify their expression profiles at the four developmental stages. This study, which is the first to describe the transcriptome of *C. comatus*, provides a foundation for future studies concerning the molecular basis of the autolysis of its fruiting bodies.

## Introduction

*Coprinus comatus* (O.F. Müll.) Pers. belongs to the Agaricaceae, Agaricales, Basidiomycota^1^, and is also known as the lawyer’s wig mushroom^2^. The fruiting bodies of this species appear in grassy fields in late summer and fall, and mainly in Asia, Europe, and North America^3–8^. *C. comatus* has medicinal properties, i.e., it can help control hypoglycemia^9^, hypolipidemia^10^, and tumor formation^5^, and can function as an antioxidant^11^. Although *C. comatus* is a well-known and well-established cultivated mushroom, its annual production has been steadily decreasing in China. For example, *C. comatus* production dropped from 189,207,012 kg in 2017 to 148,678,870 kg in 2018 (data from Edible Fungi Society of China). The main reason for this 21% decline is the autolysis that occurs as the *C. comatus* fruiting body ages.

Fungal autolysis is a natural self-degradation process that results from endogenous hydrolase activity and that involves vacuolation and disruption of the structure of organelles and cell walls^12^. During autolysis, the cell wall is degraded and the cytoplasmic contents are lost^13^. The phenomenon of autolysis is exhibited by most *Coprinus* spp.^14^, including *C. comatus*. The release of basiodiospores from the gills is accompanied by a rapid autolysis of the cap of the fruiting body leaving only the stipe intact. β-(1,3)-glucanase was believed to be the major role in the part of autolysis responsible for shedding the spores from *C. comatus*, but that the role of chitinase may increase in importance in later stages^15^.

Autolysis is thought to be the main limiting factor in the cultivation of *C. comatus*. To date, the studies of mushroom autolysis have mainly focused on storage^16–18^, and the underlying molecular processes have been infrequently assessed. Genes that encode the *exp1* transcriptional regulator^19^ and glucan hydrolases^20^ are believed to be involved in the autolysis of the fruiting bodies of the edible mushroom *Coprinopsis cinerea* (Schaeff.) Redhead. Another study found that chilling stress triggers ubiquitination-mediated autolysis in the edible mushroom *Volvariella volvacea*^21^.

Next-generation sequencing (NGS) provides a more detailed and quantitative view of gene expression, alternative splicing, and allele-specific expression than other sequencing methods^22^. Among edible mushrooms, RNA‐Seq has been used to assess gene expression in some species, such as *Pleurotus tuoliensis*^23^ and *Morchella importuna*^24^. In *Pleurotus tuoliensis*, the transcriptome of mycelia, primordia, and fruiting body were compared, and genes regulating fruiting body development were identified. In *Morchella importuna*, the study identified genes important for vegetative growth and reported that during vegetative growth enzymes involved in carbohydrate metabolism were highly expressed. Till date, there has been no report of transcriptome analysis in *C. comatus*.

In this study, we divided the growth of *C. comatus* fruiting bodies into four stages: the infant stage (I), mature stage (M), discolored stage (D), and autolysis stage (A). RNA-seq was used to screen the fruiting bodies of the four stages for the genes that might contribute to autolysis. The results could improve the storage and transportation of *C. comatus* and could provide a foundation for the genetic engineering of the species.

## Results

### Illumina sequencing and de novo assembly

**Figure 1:**
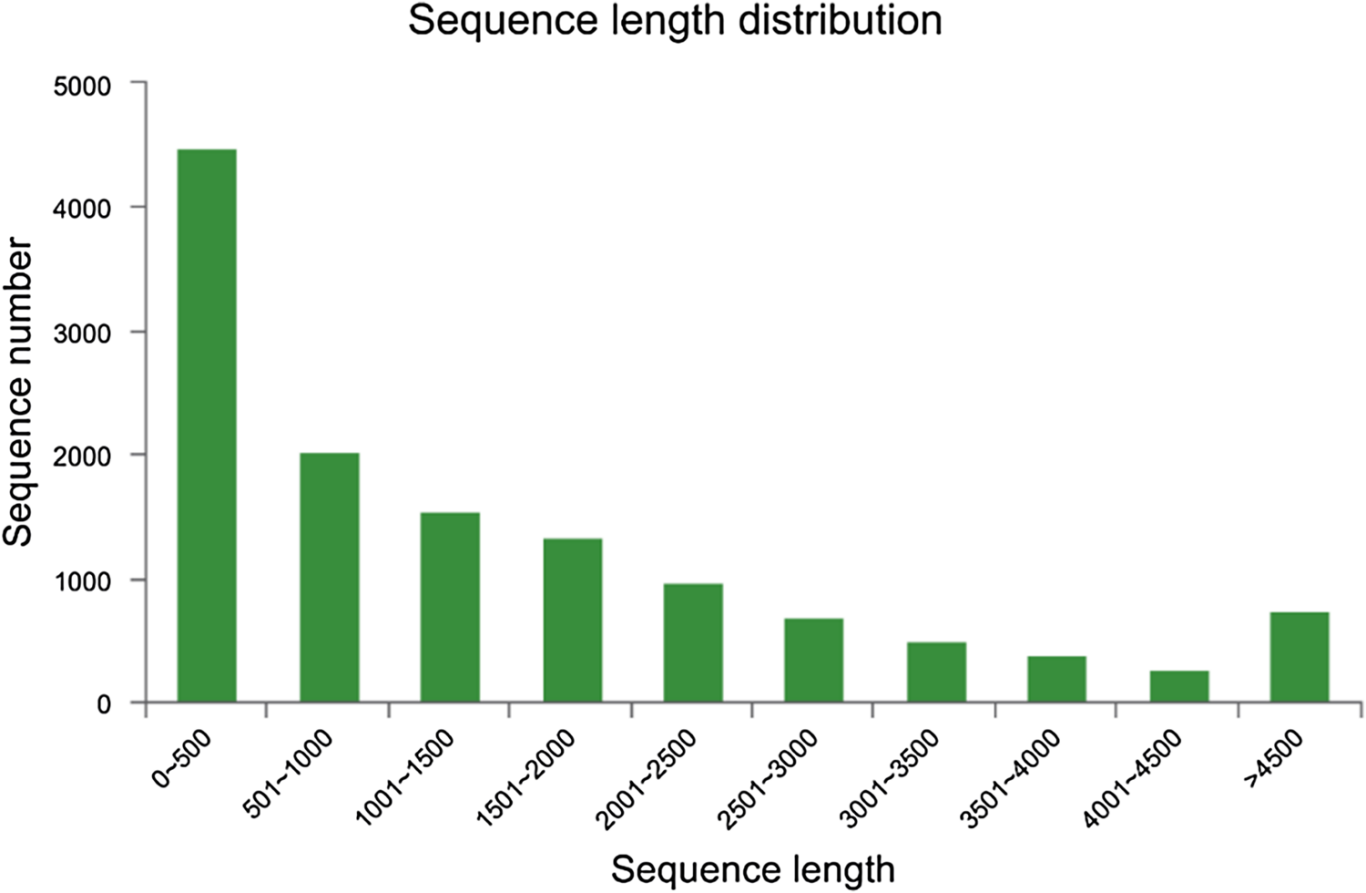
Length distribution of the final assembled unigenes. The X axis shows the sequence lengths of the unigenes, and the Y axis shows the number of unigenes.

To obtain an overview of *C. comatus* gene expression during different developmental stages, cDNA samples were prepared and sequenced using the Illumina HiSeq sequencing platform. Because a reference genome is unavailable for *C. comatus*, the transcriptome of the four stages were de novo-assembled and BLAST-annotated. A total of 752,100,560 raw reads were obtained; after removal of N reads, low quality reads, and reads with adaptor sequences, 723,022,588 clean reads remained (Table S1). In addition, Q20 and Q30 clean reads represented > 95% and > 85%, respectively, of the total number of reads in all libraries, which indicated high-quality sequencing. Finally, a total of 29,281 transcripts were further assembled into 12,946 unigenes. Among these unigenes, 6504 (50%) were 200 to 1000 bp long, and 3561 (27.5%) were > 2000 bp long (Fig. 1). The length of the shortest and longest unigene was 201 bp and 16,420 bp, respectively.

### Annotation of unique sequences

**Figure 2:**
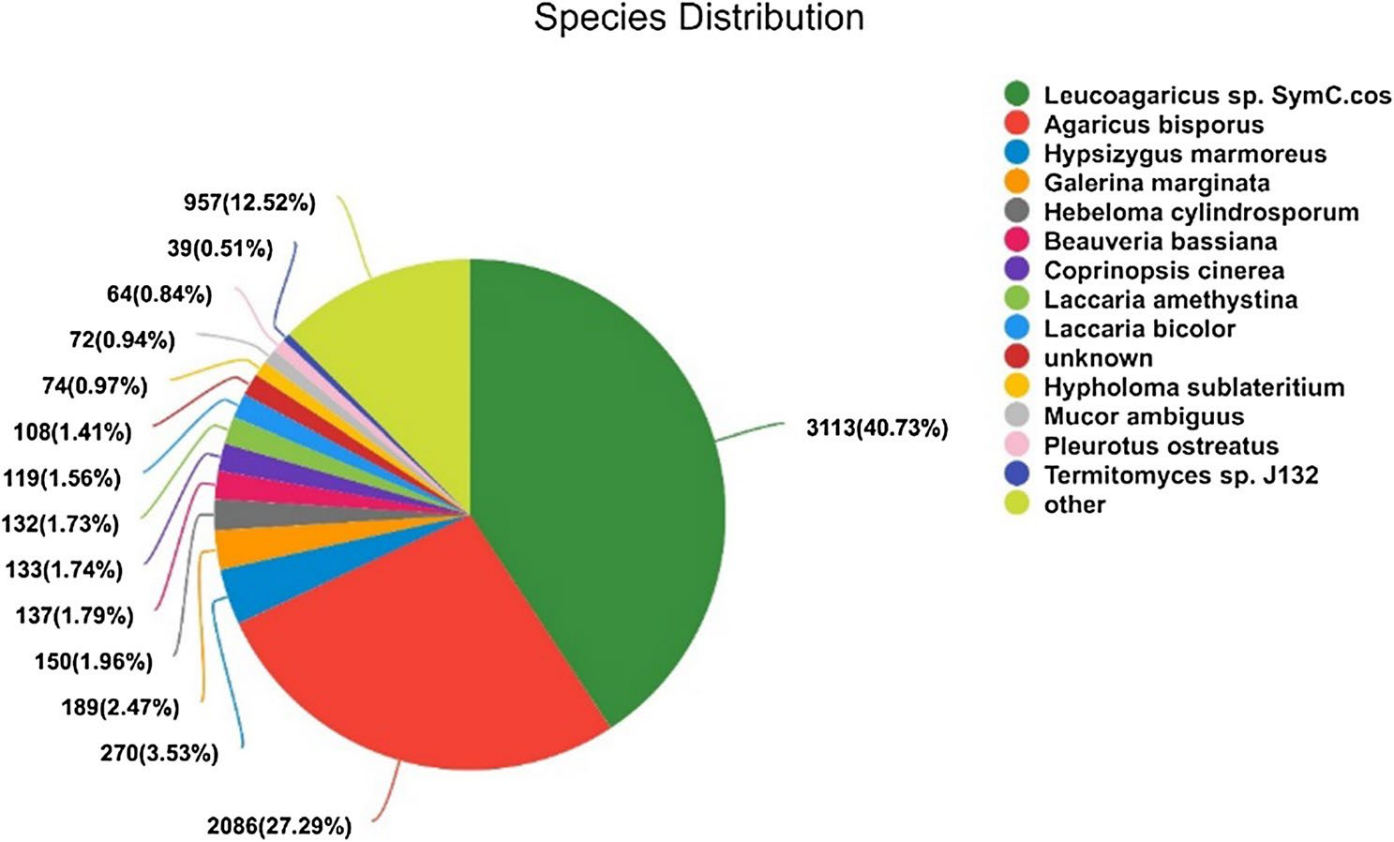
Homology (BLASTx matches) between C. comatus unigenes and unigenes of other species in the GenBank non-redundant (Nr) protein database. The first value indicates the number of matches, and the value in parenthesis indicates the percentage of all matches represented by the indicated species. Each piece of fan indicates the number of top BLAST matches against the Genbank non-redundant (Nr) protein database for various species.

In total, 8415 unigenes (65%) of *C. comatus* were annotated, and the NR database had the largest match, followed by GO, Pfam, Swiss-Prot, KEGG, and COG (Table 1). The annotated unigenes with BLASTx alignments of *C. comatus* in the NR database showed homology with other Basidiomycota (Fig. 2). Based on the NR annotation, about 5657 (73.7%) of the unigenes had E-values < 10^–30^, and 6009 (78.28%) of the unigenes showed > 60% similarity with known sequences. In terms of the species distribution of the most significant hits, a large percentage (5199, 68.01%) of the unigenes matched the sequences of *Leucoagaricus* sp. and *Agaricus bisporus*, which like *C. comatus* are in the family Agaricaceae. The *C. comatus* annotation had only 1.74% (133) matches with the DNA sequences of *Coprinopsis cinerea*, which also exhibits autolysis.

**Table 1:** Number of *C. comatus* unigenes that matched with sequences in the indicated databases (E-value < 10^–5^).

Database	Unigene number	Percentage of all unigenes (%)^a^
NR	7676	59.29
Swiss-Prot	5398	41.70
Pfam	5408	41.77
COG	1767	13.65
GO	5732	44.28
KEGG	3917	30.26
Total	12,946	

^a^Percentage of the total number of *C. comatus* unigenes (12,946) matched to the indicated database.

### GO, COG, and KEGG functional classification of *C. comatus*

**Figure 3:**
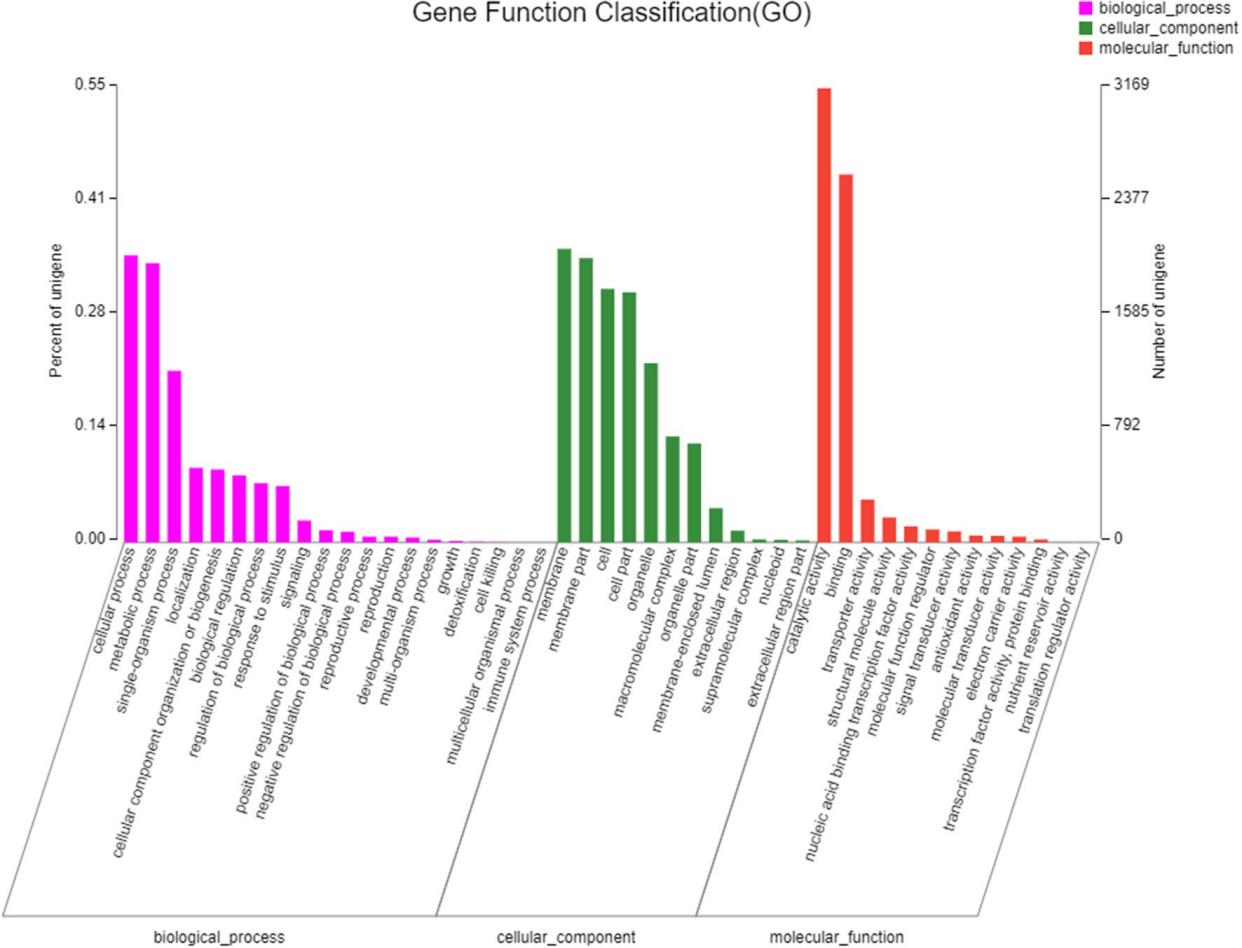
Gene ontology (GO) assignment of assembled unigenes of *C. comatus*. GO terms were processed by Blast2Go and categorized at the 2nd level under 3 main categories (biological process, cellular component, and molecular function).

A total of 5732 unigenes were categorized into 45 subcategories, which mainly included three GO categories, i.e., biological process (BP), cellular component (CC), and molecular function (MF) (Fig. 3). In the category of BP, the most dominant functional groups included “cellular process”, which had 2002 unigenes (34.9% of 5732 unigenes annotated in GO database), “metabolic process” (1947 unigenes, 34.0%), and “single-organism process” (1196 unigenes, 20.9%). In the CC category, “membrane” had 2047 unigenes (35.7%), “membrane part” had 1983 unigenes (34.6%), “cell” had 1768 unigenes (30.8%), “cell part” had 1744 unigenes (30.4%) and “organelle” had 1249 unigenes (21.8%). In the MF category, “catalytic activity” had 3169 unigenes (55.3%) and “binding” had 2567 unigenes (44.8%), which were significantly greater than the numbers of unigenes associated with the other terms (which did not exceed 10%).

**Figure 4:**
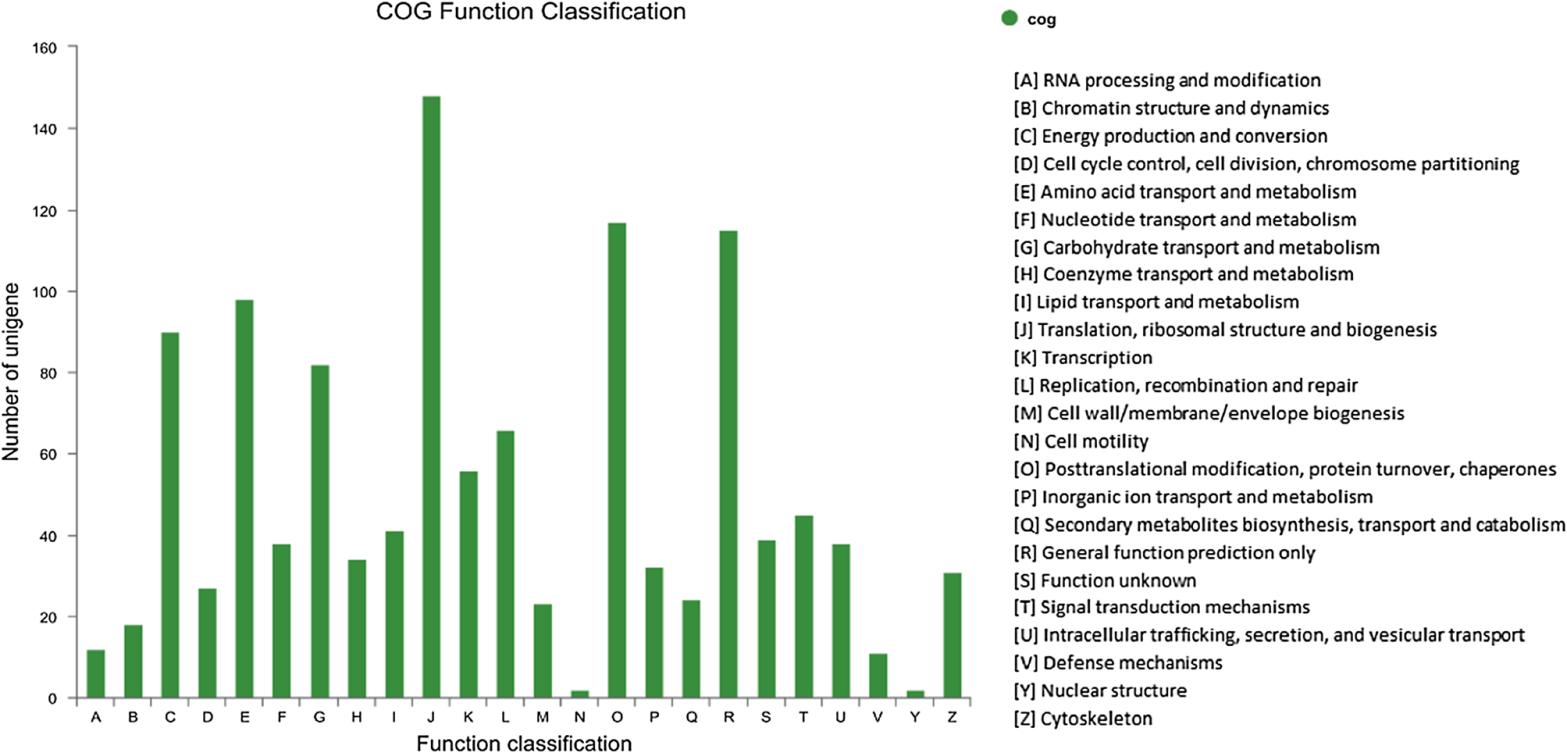
COG analysis of the unigene sequences of *C. comatus*. The Y-axis indicates the number of unigenes in a specific functional cluster. The X-axis indicates the function class.

To further evaluate the effectiveness of the annotation process and the completeness of the transcriptome, we searched the annotated sequences for unigenes involved in COGs classifications. A total of 1767 unigenes were classified into at least 24 functional groups (Fig. 4). Of these classifications, translation, ribosomal structure, and biogenesis (148, 8.4%) represented the largest group, followed by posttranslational modification, protein turnover, chaperones (117, 6.6%), general function prediction only (115, 6.2%), amino acid transport and metabolism (98, 5.5%), and energy production and conversion (90, 5.1%).

**Figure 5:**
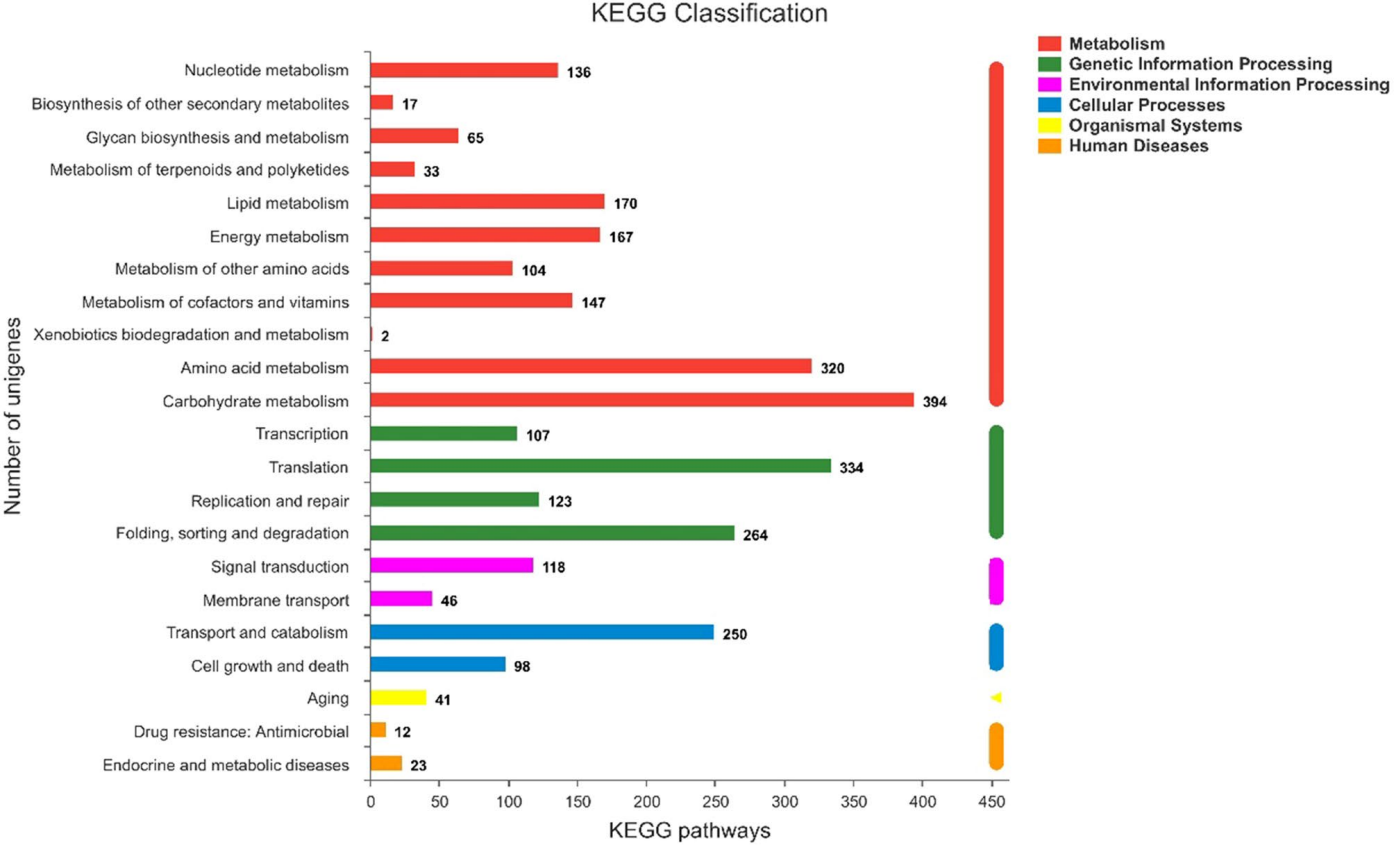
KEGG metabolic pathway of *C. comatus*. The Y-axis indicates the names of the KEGG metabolic pathways, and the X-axis indicates the number of genes.

The unigenes were mapped to reference canonical pathways in the KEGG database, and 3917 unigenes were assigned to KEGG Orthology terms, which included six main categories with 22 pathways (Fig. 5). Among the six main categories, metabolism (1555, 39.7%) was largest, followed by genetic information processing (828, 21.1%), cellular processes (348, 8.9%), environmental information processing (164, 4.2%), organismal systems (41, 1.0%), and human diseases (35, 0.9%). Overall, the top five KEGG pathways were carbohydrate metabolism (394, 10.1%); translation (334, 8.5%); amino acid metabolism (320, 8.2%); folding, sorting, and degradation (264, 6.7%); and transport and catabolism (250, 6.4%).

### Identification of differentially expressed genes (DEGs) among the four developmental stages

**Figure 6:**
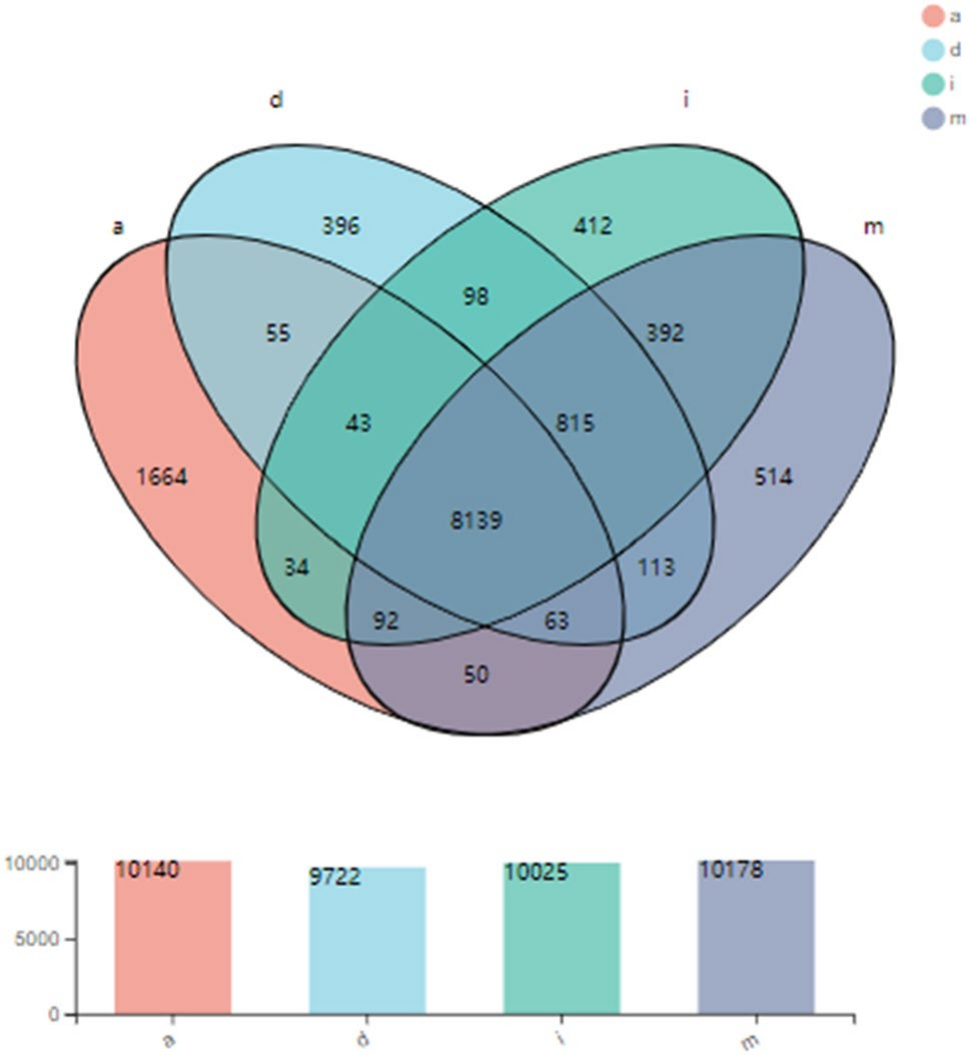
Venn diagram showing the unique and overlapping transcripts expressed in the four stages of fruiting body development in *C. comatus.*

The expression levels of genes were calculated to reveal differences in transcripts among the four stages of fruiting body development in *C. comatus* (Figs. 6 and S1). A total of 10,025, 10,178, 9722, and 10,140 unigenes were specifically expressed in the I, M, D, and A stages, respectively. A total of 8139 unigenes were shared among the four stages. A total of 392 genes were shared between stages I and M, which is higher than that shared between stages M and D (113), or between stages D and A stages (55). The results indicated that the transcripts expressed in stages I and M were more similar than the other stages. The cluster analyses of DEGs among the four stages provided similar results (Fig. S1). To gain insight into gene expression patterns during fruiting body development, we carried out a comparative genome-wide expression analysis at stages I, M, D, and A. A high number of unigenes with differential expression among the four stages were found. The resulting gene expression profiles of the four developmental stages of *C. comatus* were highly divergent. A total of 845, 4516, and 4100 DEGs were discovered when comparing M vs. I, D vs. M, and A vs. D, respectively. The TPM values were calculated, and the cluster analyses of DEGs indicated that the values for the three biological replicates in each stage were all clustered together.

### GO enrichment analysis and KEGG signaling pathway analysis of DEGs

**Figure 7:**
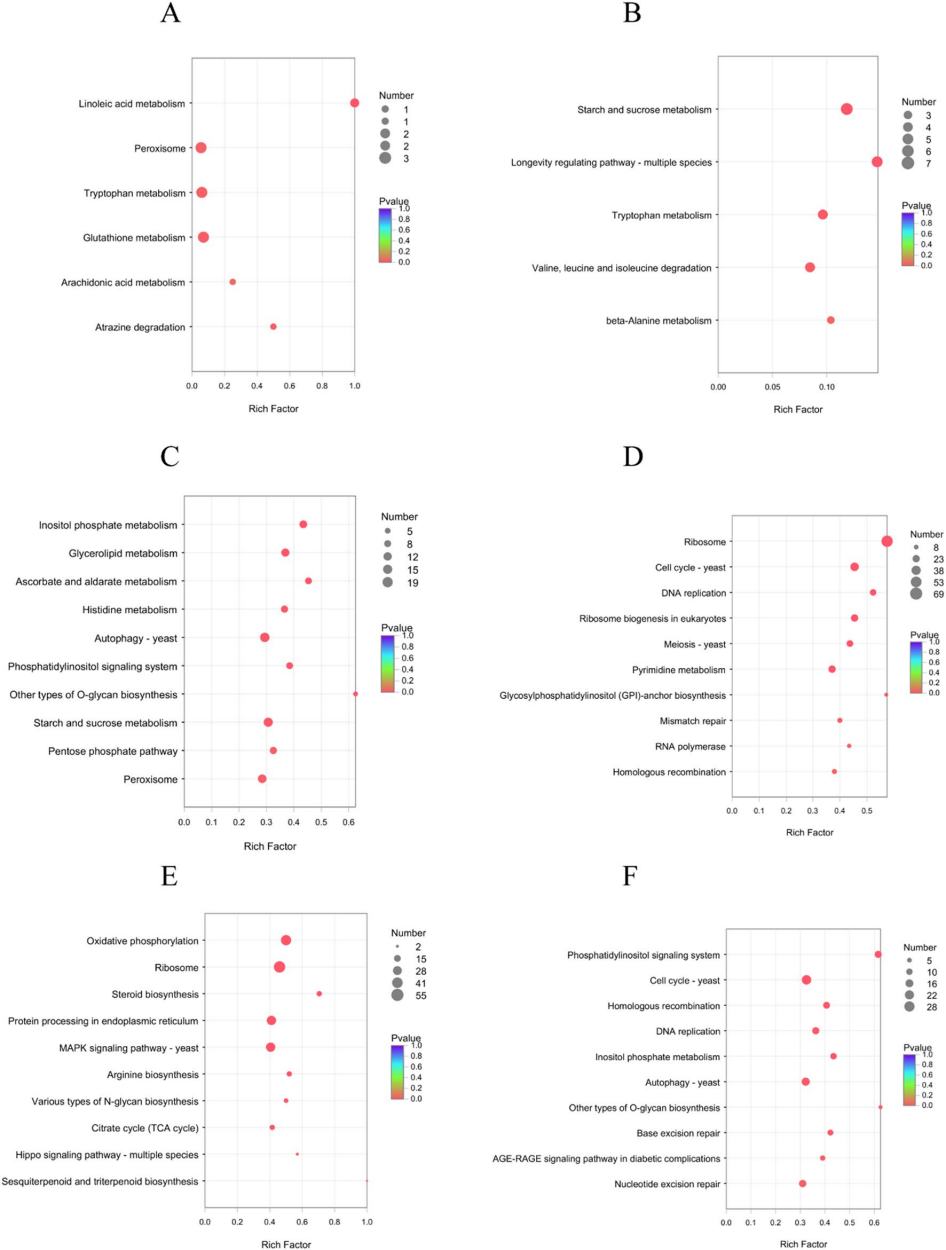
KEGG pathway enrichment analyses of the four stages of fruiting body development in *C. comatus*. (**A**) Up-regulated unigenes between stages M and I; (**B**) down-regulated unigenes between stages M and I; (**C**) up-regulated unigenes between stages D and M; (**D**) down-regulated unigenes between stages D and M; (**E**) up-regulated unigenes between stages A and D; (**F**) down-regulated unigenes between stages A and D.

The number of DEGs between M to I was the lowest and included 236 up-regulated unigenes and 609 down-regulated unigenes. For up-regulated unigenes, the main GO terms were enriched in BP and MF categories and six terms, including oxylipin biosynthetic process, oxylipin metabolic process, oxidoreductase activity, antioxidant activity, peroxidase activity, and oxidoreductase activity (acting on peroxide as acceptor) (Fig. S2A). We then performed the KEGG pathway enrichment analysis and found that the up-regulated DEGs in stage M were significantly enriched in linoleic acid metabolism, peroxisome and tryptophan metabolism pathways (Fig. 7A). The down-regulated DEGs in stage M were significantly enriched in the GO term oxidoreductase activity and KEGG pathways (e.g. starch and sucrose metabolism, longevity regulating pathway—multiple species, Figs. S2B and 7B).

In the transition from M to D, 4516 DEGs were identified, including 2201 that were up-regulated and 2315 that were down-regulated in D. GO functional enrichment analysis of the up-regulated DEGs indicated that they were mainly enriched in phosphotransferase activity, alcohol group as acceptor, protein kinase activity, and iron ion binding (Fig. S2C). KEGG enrichment analysis was used to identify the top 10 pathways of up-regulated DEGs in stage D (Fig. 7C); these included the autophagy-yeast pathway, which indicated that the process of autophagy had begun in stage D. The down-regulated DEGs in D were mainly enriched in BP and CC categories in the GO analysis (Fig. S2D). In the KEGG enrichment analysis, the down-regulated DEGs in D were mainly enriched in pyrimidine metabolism, ribosome, DNA replication, ribosome biogenesis in eukaryotes, cell cycle—yeast, and meiosis—yeast pathways (Fig. 7D).

There were 4100 DEGs identified in the comparison of stages D and A, and these included 2249 unigenes that were up-regulated and 1851 that were down-regulated in D. Based on the GO functional enrichment analysis of the up-regulated unigenes, the top 10 GO terms were significantly enriched in all the three categories (e.g. cellular component, membrane part, Fig. S2E). Based on the KEGG pathway enrichment analysis, the DEGs were significantly enriched in ten pathways (e.g. Oxidative phosphorylation, Ribosome, Fig. 7E). The morphological characteristics of fruiting bodies in stage A will show the following changes, i.e., the margin of pileus begin to form black liquid, the numerous spores form and turn black, and cell walls of hyphae are broken. These pathways may be related to the changes in the morphological characteristics. The GO functional analysis indicated that the DEGs that were down-regulated in D relative to A were mainly enriched in cellular response to DNA damage stimulus, DNA repair, and double-strand break repair (Fig. S2F). In the KEGG enrichment analysis, the down-regulated DEGs were significantly enriched in phosphatidylinositol signaling system, DNA replication, autophagy—yeast, base excision repair pathways (Fig. 7F).

### Screening of genes related to autolysis

**Figure 8:**
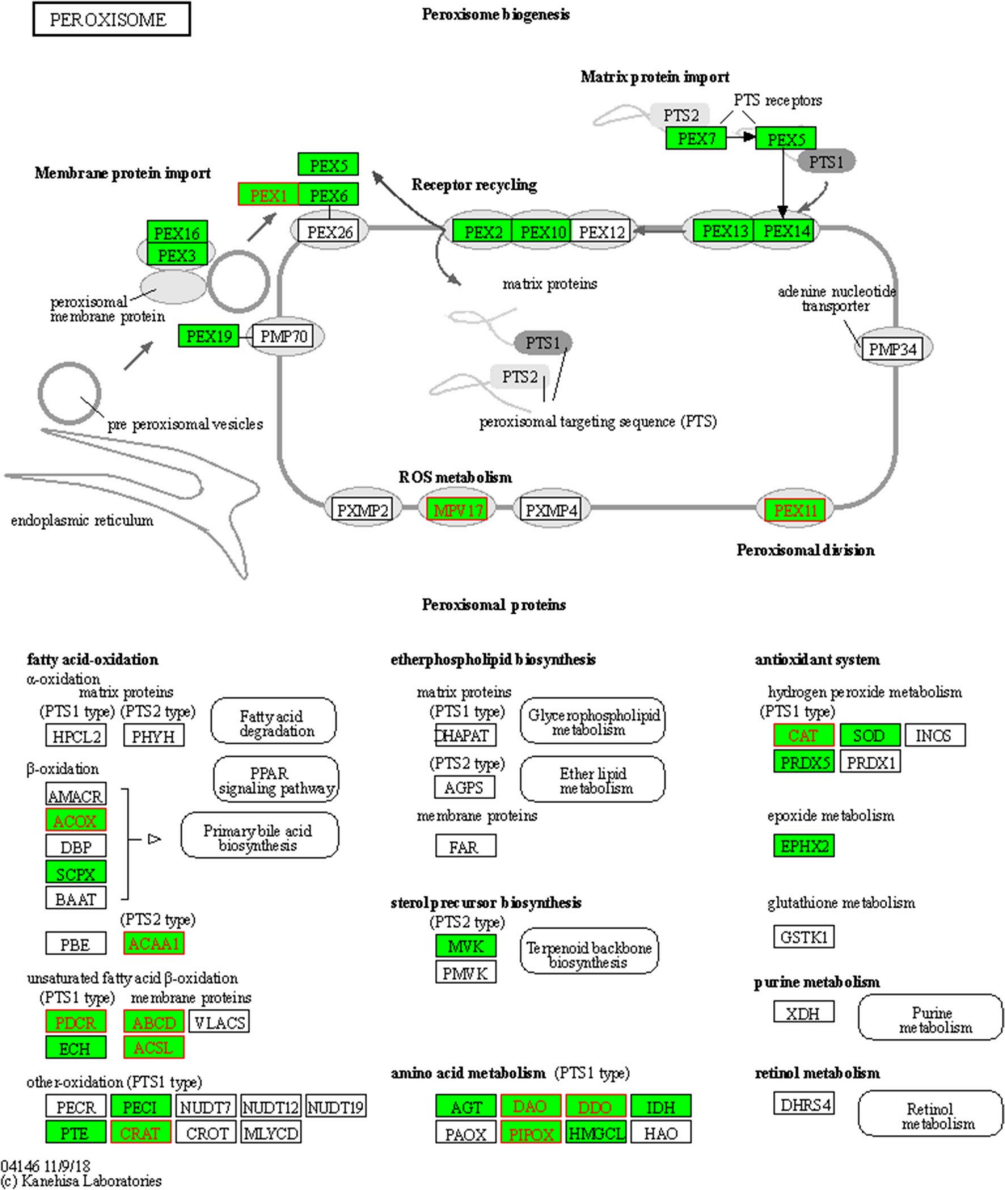
The peroxisome pathway, which might be involved in the up-regulation of genes in stage D relative to stage M of fruiting body development in *C. comatus.*

Assessment of the DEGs identified in the transition from M to D indicated that the genes in the autophagy-yeast pathway were up-regulated. We therefore focused on three pathways that involved up-regulation in the transition from stages M to D and that might be related to autolysis: autophagy—yeast^25^ (Fig. S3), peroxisome^26^ (Fig. 8), and starch and sucrose metabolism^27^ (Fig. S4).

### Validation of transcriptome data by qRT-PCR

**Figure 9:**
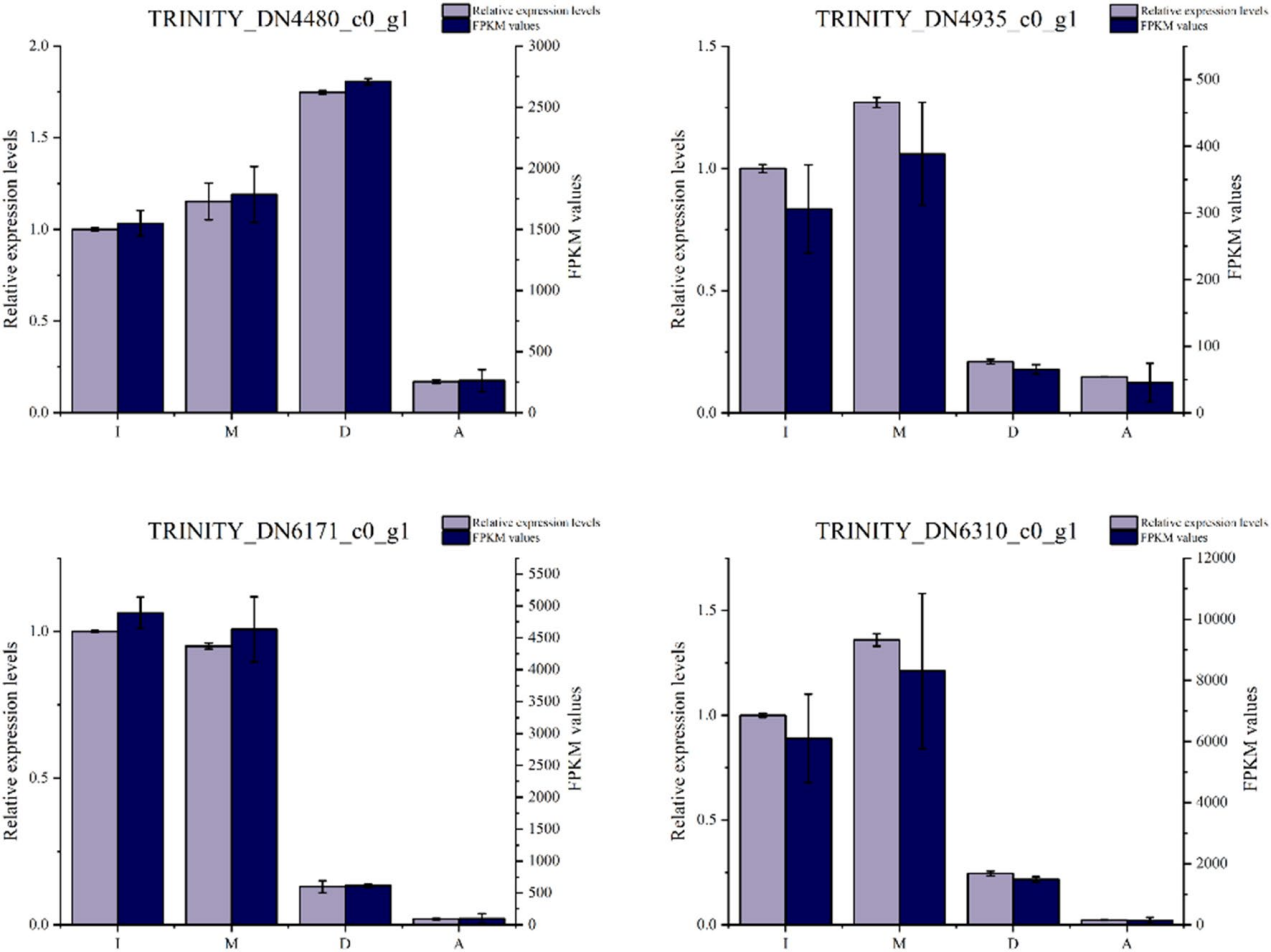
Comparison of qRT-PCR analysis of gene expression with RNA-seq data in stages I, M, D, and A of fruiting body development in *C. comatus.* The Y axis shows the relative Mrna expression levels.

To validate the expression profiles obtained from Illumina sequencing analysis, we randomly selected 20 DEGs for qRT-PCR analyses that used the same RNA samples. In all cases, the qRT-PCR data were consistent with the sequencing results (Figs. 9, S5).

## Discussion

*C. comatus* is an important edible mushroom that experiences autolysis, the molecular basis of which remains largely uncharacterized. In this study, we used Illumina RNA-sequencing to assess changes in the transcriptomes during four stages of *C. comatus* fruiting body development; these stages were, in order of fruiting body development, the infant stage (I), the mature stage (M), the discolored stage (D), and the autolysis stage (A). We then selected the genes that might be associated with autolysis; a total of 29,281 transcripts and 12,946 unigenes were obtained. We subsequently identified the DEGs of stages M and I, stages D and M, and stages A and D.

For DEGs of stages M and I, the enriched GO terms can coordinate the conversion of substances inside and outside the cell. There were many amino acid pathways in the KEGG enriched pathway, indicating that carbon metabolism and nitrogen metabolism are very active during this period. Linoleic acid metabolism and peroxisome play an important role in maintaining organism stability and biological stress in stage M. The main function of all of the enriched pathways is to provide nutrition and energy for the growth, development, and maturation of the fruiting body. These DEGs and pathways are probably related to growth of the fruiting bodies and the formation of basidiospores of *C. comatus*.

For DEGs of stages D and A, the KEGG pathway enrichment analysis showed that some pathways, like DNA replication, base excision repair, and nucleotide excision repair, significantly declined. The results showed that processes related to self-repair declined in the transition from stage D to stage A. Although the cell walls of the lamella were broken in stage A, a lot of spores formed and turned black. Analyses of the DEGs indicated that the expression of the protein *Fks2*, which is essential for sporulation^28^, and the expression of the protein *Clb1/2*, which indirectly acts on DNA damage^29^, are up-regulated in stage A. We therefore speculate that the related genes up-regulated are consistent with the variation of morphological characters in A stage.

Because the fruiting bodies in stage D began to discolor, we believed that the transition from stage M to D may be the important for autolysis. By identifying DEGs between stages D and M, we found that the pathways related to metabolic activity (like DNA replication, ribosome biogenesis, and meiosis pathways) began to decline significantly in the transition. Among the DEGs, 69 in the ribosome pathway were down-regulated. These genes encode various ribosomal proteins, and their down-regulation leads to reduced protein synthesis in stage D, suggesting that the metabolic activity of the fruiting body was lower in stage D than stage M. In contrast, the expression levels of chitinases, β-1,3-glucanases, and ubiquitin-conjugating enzyme E2 related to degradation of cell walls were significantly higher in stage D than stage M, indicating that these genes might be involved with autolysis.

Some autophagy-related genes, such as *ATG11*and *SCH9*, began to be expressed in stage D (Fig. S3). *ATG11* is essential for selective autophagy and mitophagy in yeast^30^. Mutation of *SCH9* has been reported to extend the lifespan of yeast^31^, nematodes^16,19^, flies^20^, and mice^21^. Therefore, up-regulation of these autophagy-related genes in stage D apparently indicates the onset of fruiting body senescence.

Peroxidase comprise many enzymes that are involved in various metabolic processes, such as fatty acid β-oxidation, the glyoxylic acid cycle, cholesterol synthesis, the generation and degradation of reactive oxygen species, and assimilation of methanol in yeast^32^ and plants^33^ (Fig. 8). In the current study, some genes in the peroxisome pathway were up-regulated in stage D, such as *PEX11*, *ACOX*, *ACAA1*. Among them, *PEX11* was believed to be a key factor in peroxisome proliferation and maintenance^34^. The related enzymes of peroxidase (e.g., catalase and TRINITY_DN8551_c2_g2) began to synthesized in stage D. *ACOX*, *ACAA1* promoted fatty acid decomposition and further caused membrane damage. Meanwhile, the gene expression levels of *SOD* and *PRDX5* that elimination ROS were significantly down-regulated, which further promoted the processes of cell apoptosis. In the pathway “Starch and sucrose metabolism” (Fig. S4), a total of 18 unigenes were found to be up-regulated. Among them, three unigenes (TRINITY_DN7736_c0_g1, TRINITY_DN7736_c0_g1, and TRINITY_DN8463_c0_g4) were annotated to encode glucan 1,3-beta-glucosidase (Table S2). Glucan is a primary structural component of the fungal cell wall^20^. β-1,3-glucanases contribute greatly to the autolysis of fruiting bodies^35^. For *Coprinopsis cinerea*, endo-1,3-β-glucanase, exo-1,3-β-glucanase, and 1,3-β-glucosidase may act synergistically to completely degrade the 1,3-β-glucan backbone of the cell wall during fruiting body autolysis^36^. In the present study, β-1,3-glucanases showed increased expression during stage D, and their expression level was significantly higher in stage D than in stage M. Additional research is needed regarding the effects of β-1,3-glucanases on autolysis in *C. comatus.*

In addition to β-1,3-glucanases, a number of other genes have been associated with autolysis, and these include chitinase^15^ and ubiquitinase E2 (UBE2)^21^. Chitin is a primary structural component of the fungal cell wall^37^ and is a substrate of chitinase^38^. Previous studies have shown that chitinase takes part in the rapid autolysis of the *Lentinula* fruiting body^39^. Chitinase expression was reported to increase with the maturation of fruiting bodies of coprinoid mushrooms^40^. Chitinase is thought to promote the degradation of cell walls of *Coprinopsis cinerea*^20^. The current findings are consistent with these earlier reports. In our transcriptome analysis, chitinase showed increased expression during stage D, i.e., the expression level was significantly higher in stage D than in stage M. We found three chitinase isozymes: chitinase A1, chitinase 1, and chitinase 2 (Table S3). The expression level of chitinase A1 was nearly unchanged during the transition from stage I to stage M, but it was down-regulated in stage A relative to stage D. The expression level of chitinase 2 was up-regulated from stage I to stage A. The expression level of chitinase 1 was continuously up-regulated with the development of *C. comatus* fruiting bodies, and the expression levels of TRINITY_DN8778_c0_g2 and TRINITY_DN8502_c0_g3 were nearly 20 times higher in stage A stage than in stage M. These results suggest that chitinase A1 and chitinase 2 are important in the growth of *C. comatus*, and that chitinase 1 might be important in the autolysis of *C. comatus*.

Ubiquitination is a post-translational modification pathway that mediates the growth and development of all eukaryotic species^41^. Ubiquitin-conjugating enzyme E2 (UBE2) is the main agent that selects lysines to construct ubiquitin chains^42^. The up-regulation of UBE2 was related to the autolysis of *Volvariella volvacea*^21^. In the current study, UBE2 showed increased expression during stage D, such that its expression level was significantly higher in stage D than in stage M (Table S4).

To sum up, the autolysis of *Coprinus comatus* is a complex process that involves the rupture of cell wall and membrane. Our results in this study found that expression levels of β-1,3-glucanases and chitinase in starch and sucrose metabolism were significantly up-regulated, thus leading to cell wall degradation. Meanwhile, expression levels of *ACOX* and *ACAA1* in fatty acid decomposition were also up-regulated, thus resulting in membrane damage. Furthermore, expression levels of peroxidase-encoding genes including *SOD* and *PRDX5* were down-regulated, which further promoted the processes of cell apoptosis.

## Materials and methods

### Sample preparation and morphological observations

**Figure 10:**
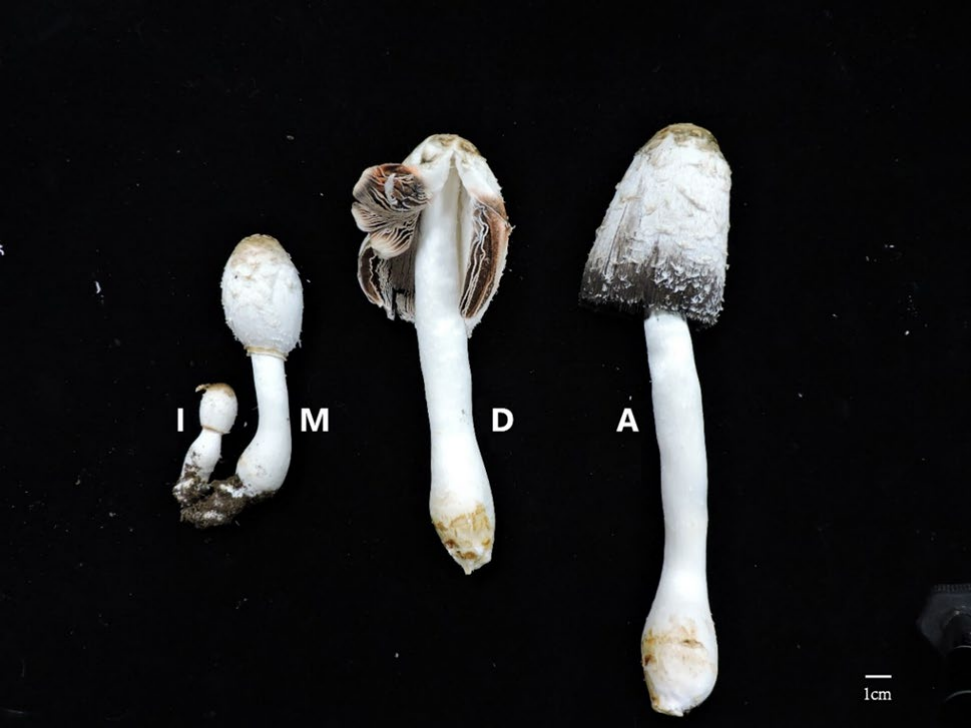
Photographs of the *C. comatus* fruiting body at four stages of development. (**A**) Infant stage; (**B**) mature stage; (**C**) discolored stage; and (**D**) autolysis stage. Scale bars 1 cm.

**Figure 11:**
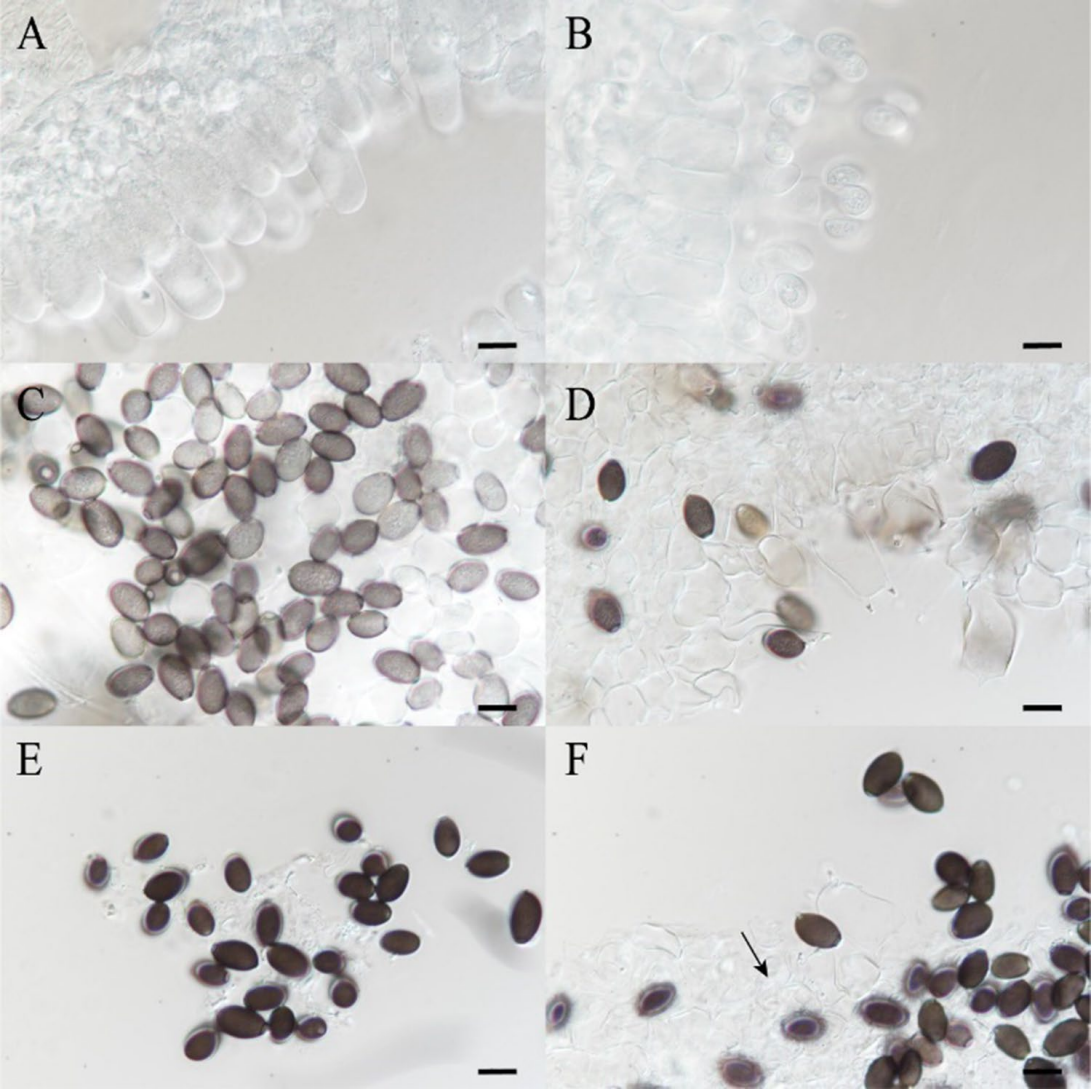
Microscopic characteristics of lamellae and basidiospores in four stages of fruiting body development in *C. comatus*. (**A**) Infant stage; (**B**) mature stage; (**C**) Basidiospores in the discolored stage; (**D**) Basidia in the discolored stage; (**E**) Basidiospores in the autolysis stage; (**F**) Basidia and hyphae of trama in the autolysis stage. Scale bars 10 μm. Note: The arrow in (**F**) indicates broken cell walls.

Strain Y13 of *C. comatus* was isolated and cultured from a sample (SYAU-FUNGI-2019003) collected from Liaoning Province, China. Both cultured mycelia and fruiting bodies were identified by the sequence of the internal transcribed spacer region of rDNA (rDNA ITS) using PCR primers ITS5/ITS4^43^. DNA sequencing and BLAST confirmation were based on the results of Matheny et al^44^. The growth of the fruiting bodies of *C. comatus* was observed continuously. As the fruiting bodies grew, their macroscopic and microscopic characteristics changed; in particular, the colors of the lamellae and basidiospores changed and cell walls changed from intact to disrupted with growth (Figs. 10 and 11). Microscopic characteristics of the fresh specimens in distilled water were examined using a light microscope (Nikon Eclipse 80i). Images were captured with a digital camera and were processed with Adobe Illustrator CC. Based on the observed morphological changes, the the growth of *C. comatus* was divided into four stages, i.e., infant stage (I), mature stage (M), discolored stage (D), and autolysis stage (A). The key morphological characteristics of the four stages are listed in Table 2. Samples (2 g) of the lamellae of the fruiting bodies at each developmental stage were frozen at -80 °C for further study.

**Table 2:** Morphological characteristics of the four stages of fruiting body development in *C. comatus*.

Stage	Lamellae	Basidiospores	Basidia	Hyphae of trama
I	White	Absent	Cell walls entire	Cell walls entire
M	White	Numerous hyaline spores	Cell walls entire	Cell walls entire
D	Pink	Numerous, nearly equal numbers of hyaline and dark spores	Cell walls entire but beginning to expand	Cell walls entire
A	Black	Numerous black spores	Cell walls expanded and broken	Cell walls broken

### RNA extraction, cDNA library construction, and Illumina sequencing

Total RNA was extracted from the lamella of fruiting bodies of different stages using TRIzol® Reagent according to the manufacturer’s instructions (Invitrogen, USA), and genomic DNA was removed using DNase I (TaKara). We preformed three replicates for each group. The integrity and purity of the extracted RNA was determined with a 2100 Bioanalyser (Agilent Technologies, Inc., Santa Clara CA, USA) and the extracted RNA was quantified with an ND-2000 (NanoDrop Thermo Scientific, Wilmington, DE, USA). Only high-quality RNA samples (OD260/280 = 1.8–2.2, OD260/230 ≥ 2.0, RIN ≥ 8.0, 28S:18S ≥ 1.0, > 1 μg) were used to construct sequencing libraries.

RNA purification, reverse transcription, library construction, and sequencing were performed at Shanghai Majorbio Bio-pharm Biotechnology Co., Ltd. (Shanghai, China) according to the manufacturer’s instructions (Illumina, San Diego, CA). The *C. comatus* RNA-seq transcriptome libraries were prepared using the Illumina TruSeqTM RNA sample preparation Kit (San Diego, CA). Poly(A) mRNA was purified from total RNA using oligo-dT-attached magnetic beads and was then fragmented with fragmentation buffer. With these short fragments as templates, double-stranded cDNA was synthesized using a SuperScript double-stranded cDNA synthesis kit (Invitrogen, CA) with random hexamer primers (Illumina). The synthesized cDNA was then subjected to end-repair, phosphorylation, and ‘A’ base addition according to Illumina’s library construction protocol. Libraries were size-selected for cDNA target fragments of 200–300 bp on 2% Low Range Ultra Agarose followed by PCR amplification using Phusion DNA polymerase (New England Biolabs, Boston, MA) for 15 PCR cycles. After they were quantified by TBS380, two RNAseq libraries were sequenced in single lanes on an Illumina Hiseq xten/NovaSeq 6000 sequencer (Illumina, San Diego, CA) for 2 × 150-bp paired-end reads.

### De novo assembly and annotation

The raw paired-end reads were trimmed and quality controlled by SeqPrep (https://github.com/jstjohn/SeqPrep) and Sickle (https://github.com/najoshi/sickle) with default parameters. The clean data from the samples were used to conduct de novo assembly of transcripts with Trinity^45^. All of the assembled transcripts were searched against the NCBI protein nonredundant (NR), Swiss-Prot, Cluster of Orthologous Groups databases (COG), and Genomes pathway (KEGG) databases using BLASTX. This was done to identify the proteins in order to determine their function annotations; the cut-off E-value was set at < 1.0 × 10^−5^. HMMER3.0 was then used to annotate the unigenes in the Pfam database. BLAST2GO^46^ was used to obtain Gene Ontology (GO) annotations of uniquely assembled transcripts for describing biological processes, molecular functions, and cellular components. Metabolic pathway analysis was performed using the Kyoto Encyclopedia of Genes and Genomes^47^.

### Analysis of differentially expressed genes (DGEs)

To identify DEGs between two different samples, the expression level of each transcript was calculated according to the transcripts per million reads (TPM) method. RSEM^48^ was used to quantify gene abundances. Differential expression analysis was performed using the DESeq2^49^, and genes with absolute fold change > 2 and Q values ˂ 0.05 were identified as significant DEGs; genes with absolute fold change. In addition, functional-enrichment analysis including GO and KEGG were performed to identify which DEGs were significantly enriched in GO terms and metabolic pathways at a Bonferroni-corrected P-value ≤ 0.05 compared with the whole-transcriptome background. GO functional enrichment and KEGG pathway analysis were conducted with Goatools^50^ and KOBAS^51^.

### Quantitative real-time PCR (qRT-PCR) validation

Amplification was performed using 0.5 μL of the primers, 5 μL of 2 × SuperReal PreMix Plus (SYBR Green, TIANGEN), 3.7 μL of RNase-Free ddH_2_O (TIANGEN), and 0.8 μL of cDNA in a final volume of 10 μL. The cycling parameters were 95 °C for 15 min followed by 30 cycles of 95 °C for 10 s, 60 °C for 20 s, and 72 °C for 30 s. Three independent biological replicates were performed for each gene tested in real-time PCR reactions. The relative gene expression was analyzed using the 2^−ΔΔCT^ method. The expression of the 18S ribosomal RNA gene (unigene ID: TRINITY_DN8501_c1_g1, 84.4% similarity) was stable in the four developmental stages based on the RNA-seq data; this gene was therefore used as the internal reference in this study. We randomly selected 20 DEGs for qRT-PCR analysis. The primers used in this study are listed in Table S5.

## Supplementary Information

Supplementary Information.
