# Supplementary material for: Transcriptome analysis of genes associated with autolysis of Coprinus comatus
Source: Sci Rep. 2022 Feb 15;12:2476. doi: 10.1038/s41598-022-06103-z (PMC8847592; doi:10.1038/s41598-022-06103-z)
Supplement: Supplementary file 1 — Supplementary Information. [file 41598_2022_6103_MOESM1_ESM.docx]

Table S1. Summary of *C. comatus* transcriptome sequencing, assembly, and annotation.

| Sample | Raw reads | Clean reads | Clean bases | Error rate (%) | Q20(%) | Q30(%) | GC content (%) | Total mapped | |
| --- | --- | --- | --- | --- | --- | --- | --- | --- | --- |
| I_1 | 68391126 | 65754428 | 9.75G | 0.0124 | 98.27 | 94.89 | 48.05 | 22750548 | (69.20%) |
| I_2 | 64140950 | 61741866 | 9.15G | 0.0123 | 98.3 | 94.99 | 47.89 | 21711752 | (70.33%) |
| I_3 | 65703758 | 63087606 | 9.36G | 0.0125 | 98.22 | 94.78 | 48.05 | 21871984 | (69.34%) |
| M_1 | 65147740 | 62603524 | 9.28G | 0.0124 | 98.25 | 94.84 | 47.69 | 22566616 | (72.09%) |
| M_2 | 71934920 | 69185840 | 10.26G | 0.0124 | 98.28 | 94.93 | 47.92 | 24725570 | (71.48%) |
| M_3 | 66892488 | 63895866 | 9.46G | 0.0129 | 98.05 | 94.33 | 47.96 | 22519822 | (70.49%) |
| D_1 | 65687062 | 63320304 | 9.40G | 0.0122 | 98.34 | 95.1 | 47.01 | 22048355 | (69.64%) |
| D_2 | 60967900 | 58743744 | 8.72G | 0.0122 | 98.33 | 95.07 | 47.07 | 20468285 | (69.69%) |
| D_3 | 63227044 | 60840750 | 9.02G | 0.0124 | 98.27 | 94.92 | 46.99 | 21411547 | (70.39%) |
| A_1 | 57196868 | 55010626 | 8.15G | 0.0124 | 98.27 | 94.9 | 48.1 | 17529150 | (63.73%) |
| A_2 | 51306028 | 49296848 | 7.32G | 0.0123 | 98.29 | 94.96 | 48.13 | 13293205 | (53.93%) |
| A_3 | 51504676 | 49541186 | 7.35G | 0.0123 | 98.3 | 94.97 | 48.13 | 13959906 | (56.36%) |
| Total | 752100560 | 723022588 | 107.22G | - | - | - | - | - |  |

Table S2. Gene ID of glucanase-related genes detected in the four stages of fruiting body development in *C. comatus*.

| Gene ID | I | M | D | A | Encoded protein |
| --- | --- | --- | --- | --- | --- |
| TRINITY_DN8574_c2_g2 | 14.9 | 16.1 | 23.8 | 7.9 | glucan 1,3-beta-glucosidase |
| TRINITY_DN7410_c1_g1 | 21.0 | 18.1 | 45.3 | 1.8 | putative glucan 1,3-beta-glucosidase A |
| TRINITY_DN7736_c0_g1 | 150.9 | 140.9 | 347.1 | 155.5 | putative glucan 1,3-beta-glucosidase D |
| TRINITY_DN8171_c0_g1 | 20.0 | 4.9 | 139.7 | 6.7 | glucan 1,3-beta-glucosidase 3 |
| TRINITY_DN6276_c0_g1 | 39.3 | 64.3 | 123.8 | 8.0 | putative glucan endo-1,3-beta-glucosidase btgC |
| TRINITY_DN8007_c0_g4 | 12.0 | 3.2 | 5.8 | 0.3 | putative glucan 1,3-beta-glucosidase D |
| TRINITY_DN7659_c1_g1 | 4.0 | 4.5 | 17.3 | 0.5 | putative glucan 1,3-beta-glucosidase A |
| TRINITY_DN8463_c0_g4 | 13.0 | 3.3 | 5.4 | 0.6 | putative glucan 1,3-beta-glucosidase D |
| TRINITY_DN6514_c0_g1 | 185.2 | 60.1 | 289.7 | 38.1 | probable glucan 1,3-beta-glucosidase D |
| TRINITY_DN7235_c0_g1 | 163.1 | 275.1 | 616.2 | 868.1 | glucan 1,3-beta-glucosidase |
| TRINITY_DN7058_c0_g10 | 366.6 | 212.5 | 967.8 | 17.1 | putative glucan endo-1,3-beta-glucosidase btgC |

| Table S3. ID of related chitinase-encoding genes in the four stages of fruiting body development in *C. comatus*. | | | | | |
| --- | --- | --- | --- | --- | --- |
| Gene ID | I | M | D | A | Function |
| TRINITY_DN6171_c0_g1 | 4895.3 | 4635.0 | 614.7 | 99.0 | Chitinase A1 |
| TRINITY_DN4935_c0_g1 | 306.0 | 388.7 | 65.3 | 45.3 | Chitinase A1, partial |
| TRINITY_DN7718_c1_g1 | 3616.3 | 3313.3 | 887.7 | 511.3 | Chitinase A1 |
| TRINITY_DN6310_c0_g1 | 6100.7 | 8304.3 | 1491.0 | 141.3 | Chitinase A1 |
| TRINITY_DN8502_c0_g1 | 223.0 | 296.0 | 501.3 | 15769.0 | Chitinase 1 |
| TRINITY_DN8778_c0_g2 | 695.7 | 1488.3 | 27054.0 | 112343.3 | Chitinase 1 |
| TRINITY_DN8502_c0_g3 | 201.3 | 304.3 | 6237.7 | 291724.0 | Chitinase 1 |
| TRINITY_DN8338_c1_g1 | 4531.3 | 6219.3 | 10149.3 | 9540.0 | Chitinase 2 |

Table S4. Gene ID of UBE2-related genes detected in the four stages of fruiting body development in *C. comatus*.

| Gene ID | I | M | D | A | Encoded protein |
| --- | --- | --- | --- | --- | --- |
| TRINITY_DN4480_c0_g1 | 1550.0 | 1786.3 | 2708.7 | 263.3 | ubiquitin-conjugating enzyme E2 S |
| TRINITY_DN6390_c0_g1 | 2488.0 | 2288.0 | 2535.7 | 426.0 | ubiquitin-conjugating enzyme E2 1 |
| TRINITY_DN8106_c1_g1 | 7257.0 | 8417.7 | 10985.3 | 2407.0 | E2 ubiquitin-conjugating enzyme 5 |
| TRINITY_DN8566_c0_g1 | 1559.7 | 1466.3 | 2941.3 | 240.7 | E2 ubiquitin-conjugating enzyme 25 |

Table S5. The primers used in this study.

| Gene ID | 5’-3’ |
| --- | --- |
| TRINITY_DN4480_c0_g1-F | ATGGTATCGCCTGTACAT |
| TRINITY_DN4480_c0_g1-R | TCATAGTCTCTTCAACGC |
| TRINITY_DN4935_c0_g1-F | ATGAAGTCATTCGCTAT |
| TRINITY_DN4935_c0_g2-R | CTACAGGTTCATTGTAGCA |
| TRINITY_DN6171_c0_g1-F | AGGTGGTTTCGAGTGCCTTC |
| TRINITY_DN6171_c0_g1-R | GTAAACCTCGAGGCACCAGT |
| TRINITY_DN6276_c0_g1-F | ACAGTCTCGGTAACGCTTGG |
| TRINITY_DN6276_c0_g1-R | CGTCAGGATTGGGCTGAGTT |
| TRINITY_DN6310_c0_g1-F | TCAAGTTCGGGTTCAACGCA |
| TRINITY_DN6310_c0_g1-R | ACCTGAACCTCCGCATTTCT |
| TRINITY_DN6390_c0_g1-F | ATGCCATCTCTTCCTCCAC |
| TRINITY_DN6390_c0_g1-R | TTATTTCAGAAGTTCCTC |
| TRINITY_DN7102_c3_g2-F | TGACGTACGGCCTTTCTCAC |
| TRINITY_DN7102_c3_g2-R | GCCCTCCGTCGTCTTTACTC |
| TRINITY_DN7410_c1_g1-F | ATGAACATGGAAAACTTC |
| TRINITY_DN7410_c1_g1-R | TTACTGGAAGAGCTTTGC |
| TRINITY_DN7718_c1_g1-F | ATGACGTACGGCCTCTTCTG |
| TRINITY_DN7718_c1_g1-R | TGCATTTGTTTCTTGGCGGC |
| TRINITY_DN7736_c0_g1-F | CCGATGTACTACGAGAAC |
| TRINITY_DN7736_c0_g1-R | TTAAATCTCGACTGAAGC |
| TRINITY_DN7782_c0_g1-F | GGAACGCAGACACTGGAGAA |
| TRINITY_DN7782_c0_g1-R | CCTCCTGTTGCGAGGATTGT |
| TRINITY_DN8007_c0_g4-F | ATGGAACTCGACCAGGGC |
| TRINITY_DN8007_c0_g4-R | GGCATTGGCAAGTCCCAT |
| TRINITY_DN8106_c1_g1-F | ATGGATCTAACACTATCTC |
| TRINITY_DN8106_c1_g1-R | TCAATTAGCATACTTCTT |
| TRINITY_DN8171_c0_g1-F | ATGCGCAAGTTGTTCGCC |
| TRINITY_DN8171_c0_g1-R | TCAAGAGGACCCATATAC |
| TRINITY_DN8361_c0_g1-F | GAAATTCGACGTGCCATCCG |
| TRINITY_DN8361_c0_g1-R | GCGCCCATGTCGAGAGAATA |
| TRINITY_DN8502_c0_g1-F | GAAGAAATCTGCTGCTGCTA |
| TRINITY_DN8502_c0_g1-R | ACCACCACAAGCACTGATTG |
| TRINITY_DN8566_c0_g1-F | TTTACAGGCTGCGTGCCATA |
| TRINITY_DN8566_c0_g1-R | GAAAAACCAGCGGGTAGGGA |
| TRINITY_DN8574_c2_g2-F | TTGGAAATGGGTCCAGTGGG |
| TRINITY_DN8574_c2_g2-R | ACAACTTTCATTTGCCGCCC |
| TRINITY_DN8621_c0_g2-F | ATGACAGGCAGTTTTCTT |
| TRINITY_DN8621_c0_g2-R | CTAAGCACCACGATAACC |
| TRINITY_DN8778_c0_g2-F | ATGTTCTGGGAGCTCTCG |
| TRINITY_DN8778_c0_g2-R | TTAGCAGGCACCACTGTC |


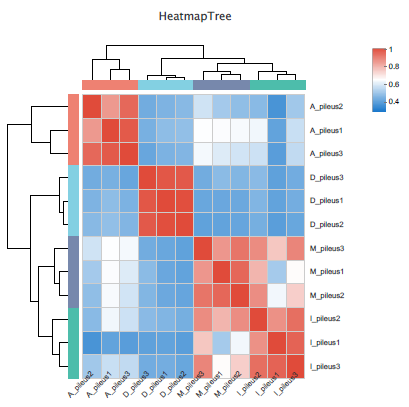


Figure S1. Cluster analyses of DEGs among the four stages of fruiting body development in *C. comatus.* The expression levels were log10 transformed; high levels of expression are indicated in red, and low levels of expression are indicated in blue.


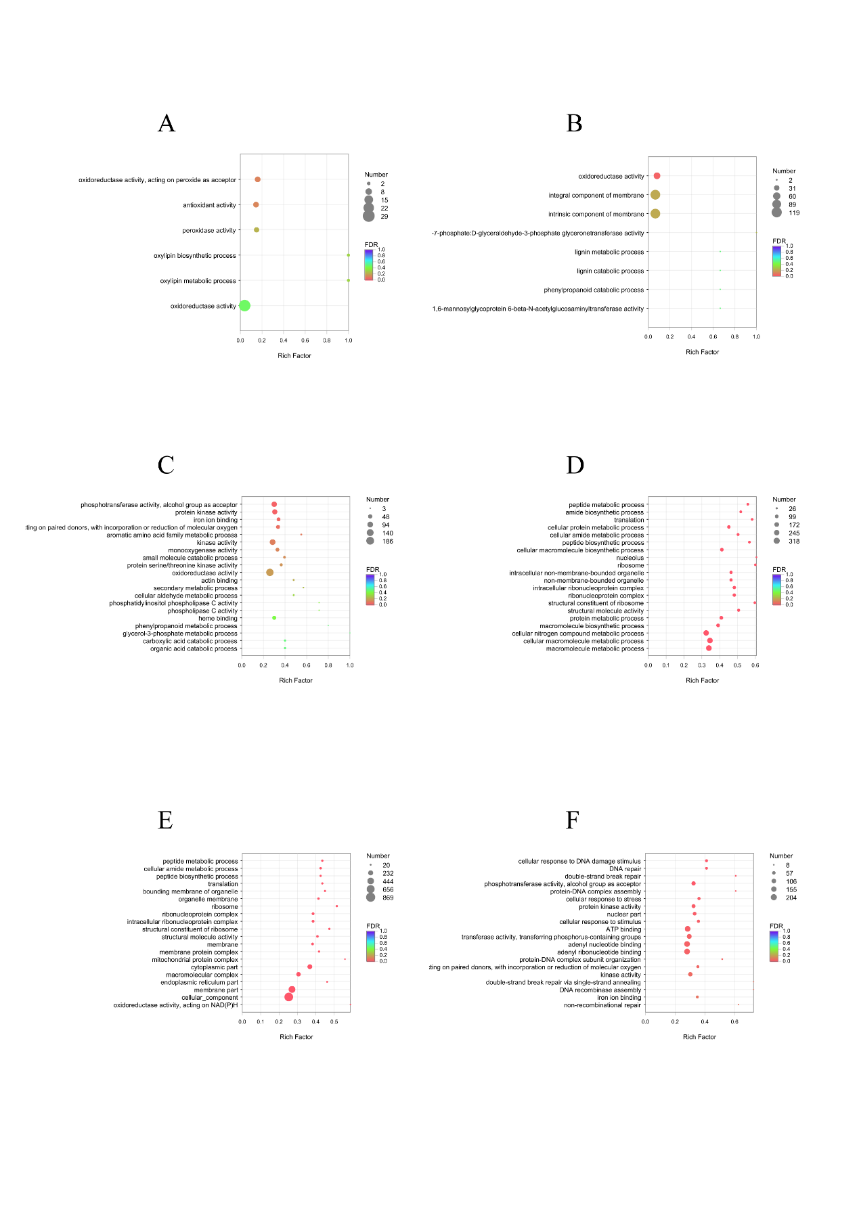


Figure S2. GO functional enrichment analyses of the four stages of fruiting body development in *C. comatus*. **A**: up-regulated unigenes between stages M and I; **B**: down-regulated unigenes between stages M and I; **C**: up-regulated unigenes between stages D and M; D: down-regulated unigenes between stages D and M; E: up-regulated unigenes between stages A and D; F: down-regulated unigenes between stages A and D.


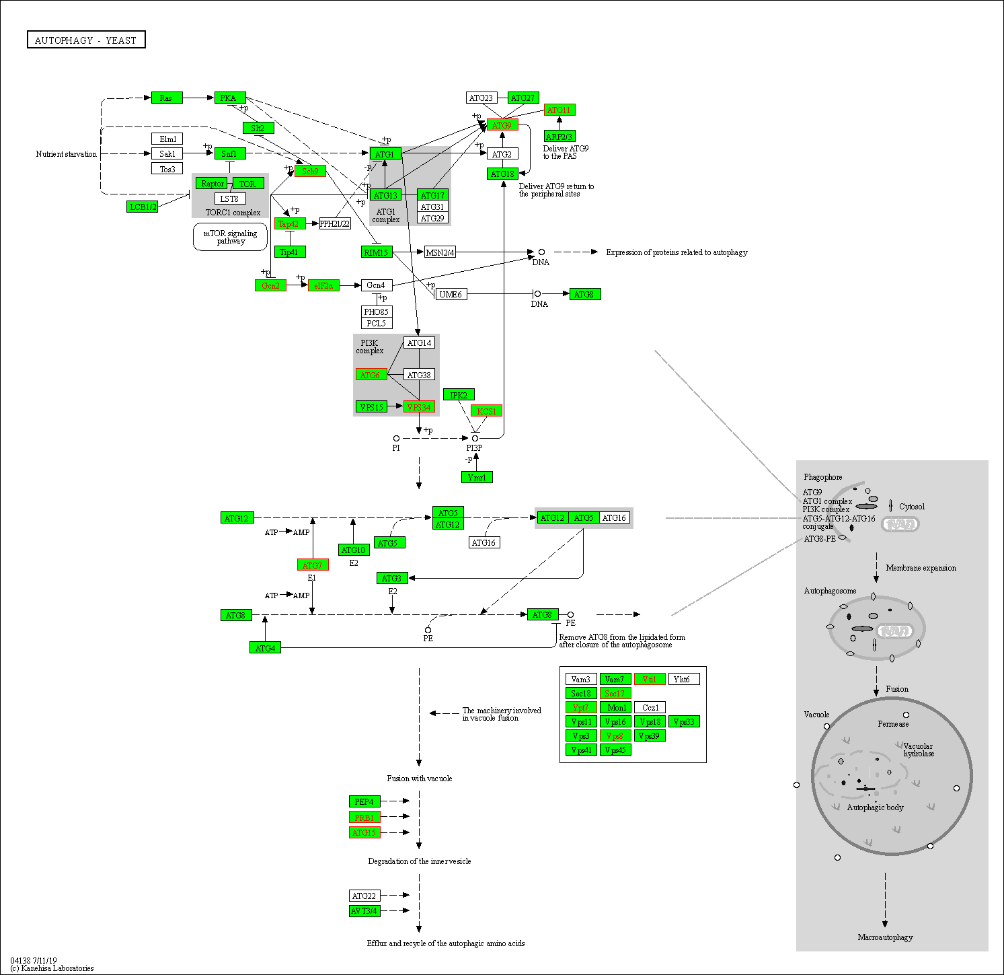


Figure S3. The autophagy–yeast pathway, which might be involved in the up-regulation of genes in stage D relative to stage M of fruiting body development in *C. comatus.*


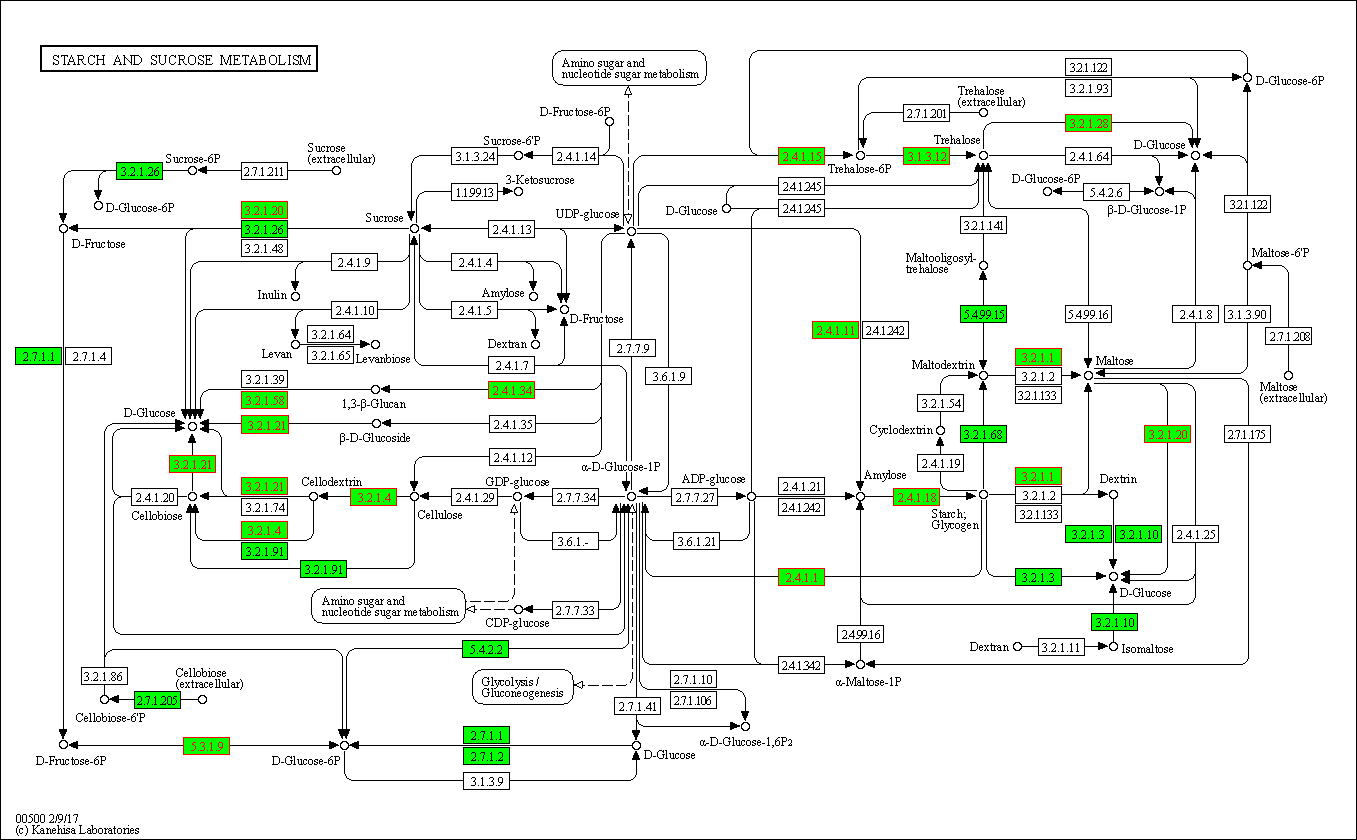


**Figure S4.** The starch and sucrose metabolism pathway, which might be involved in the up-regulation of genes in stage D relative to stage M of fruiting body development in *C. comatus.*


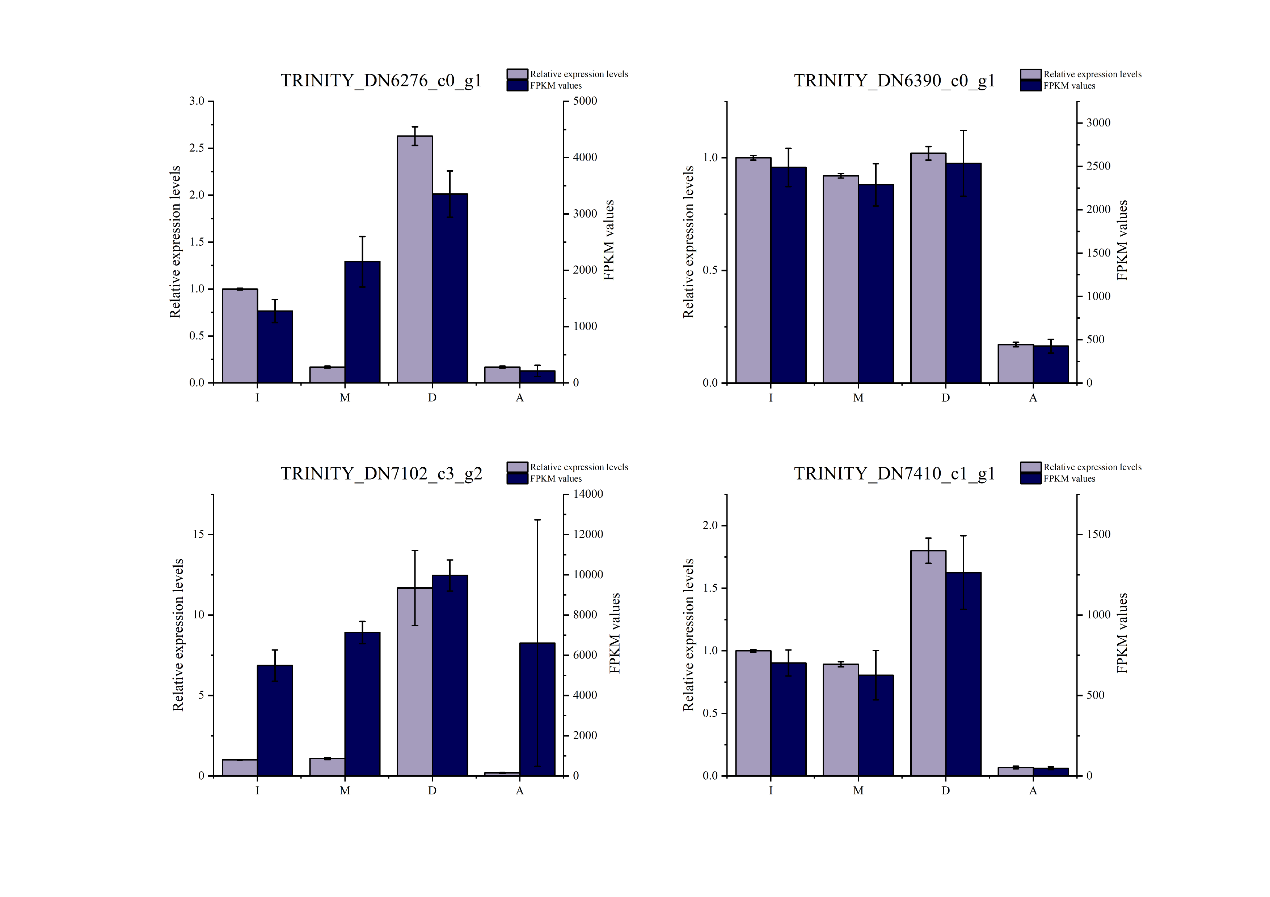


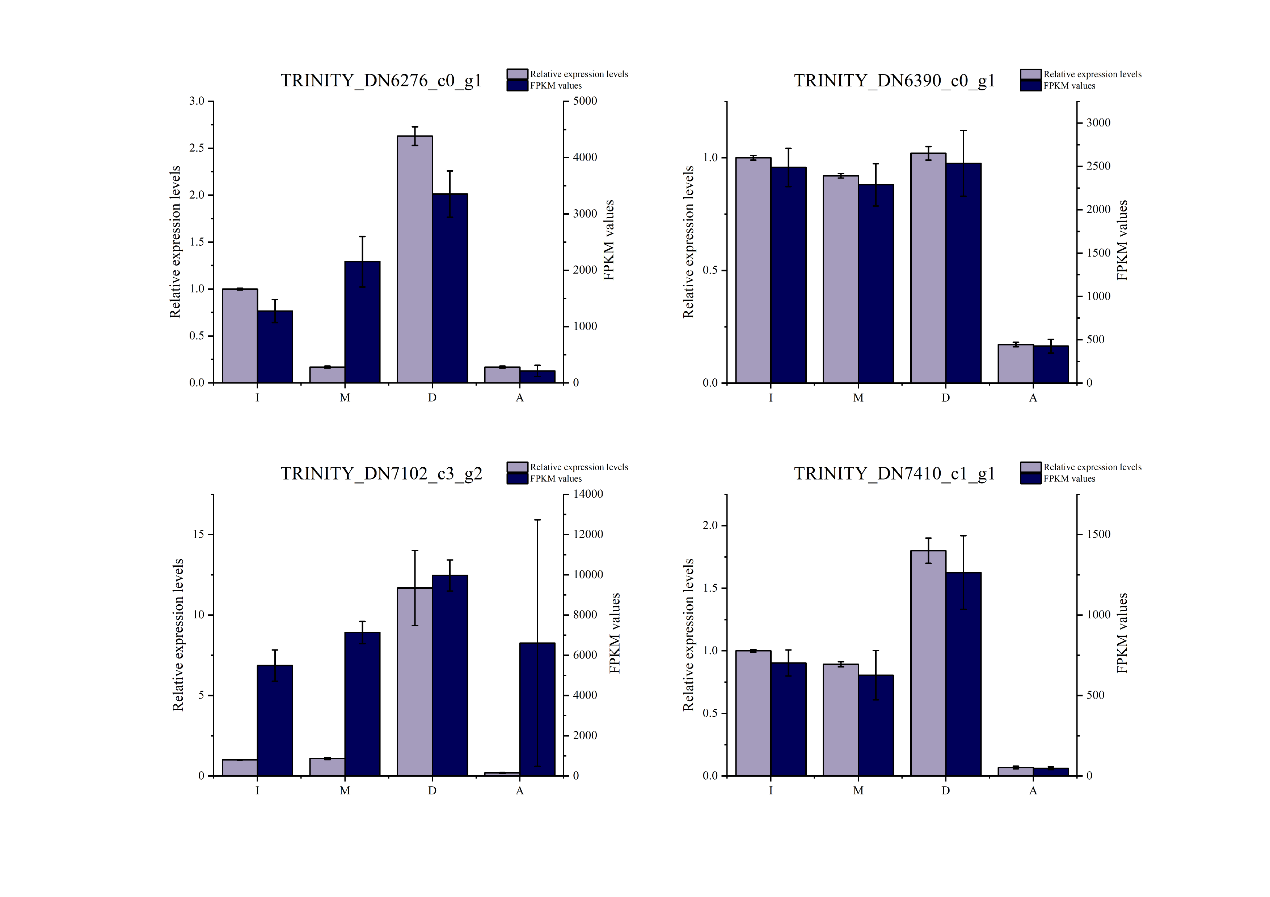


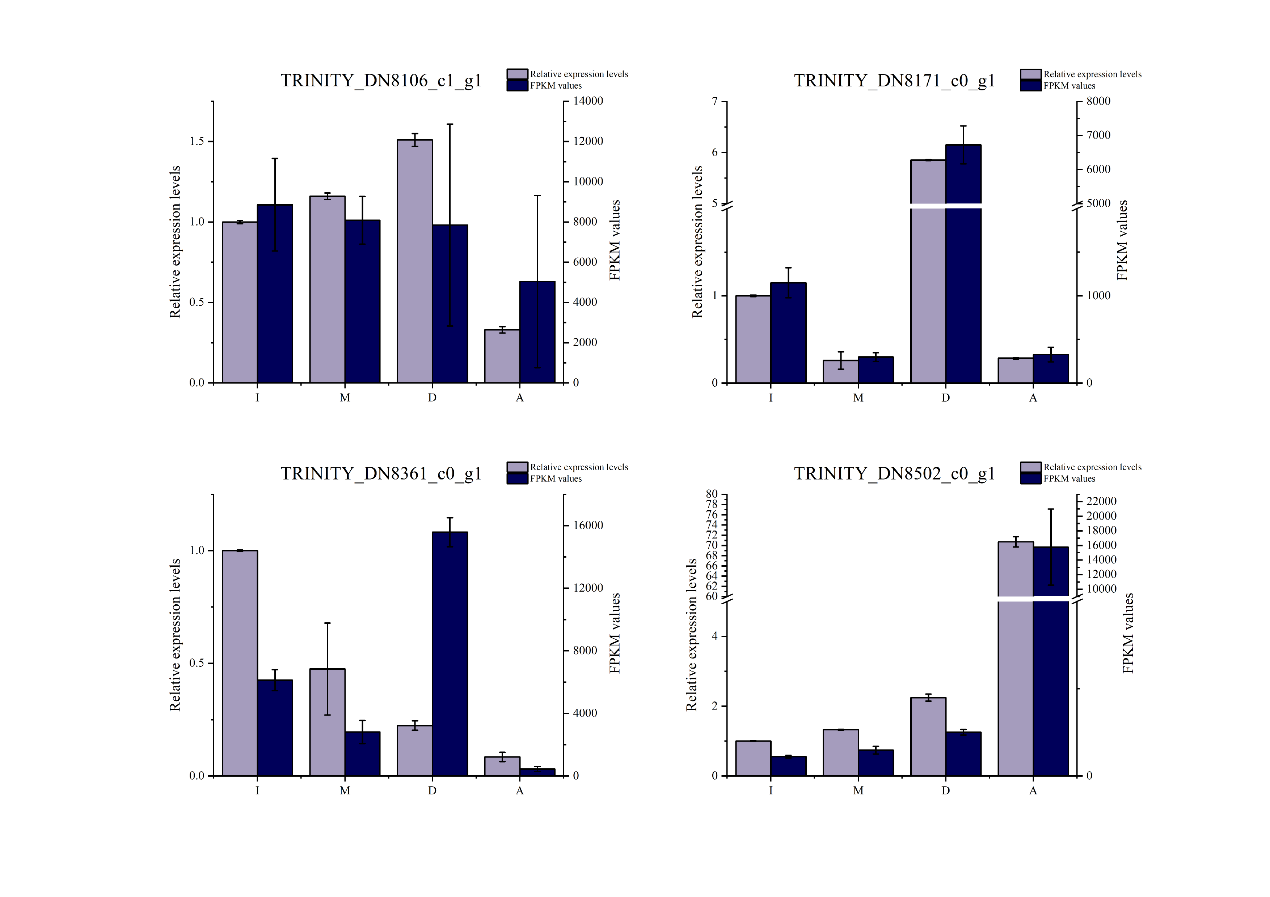


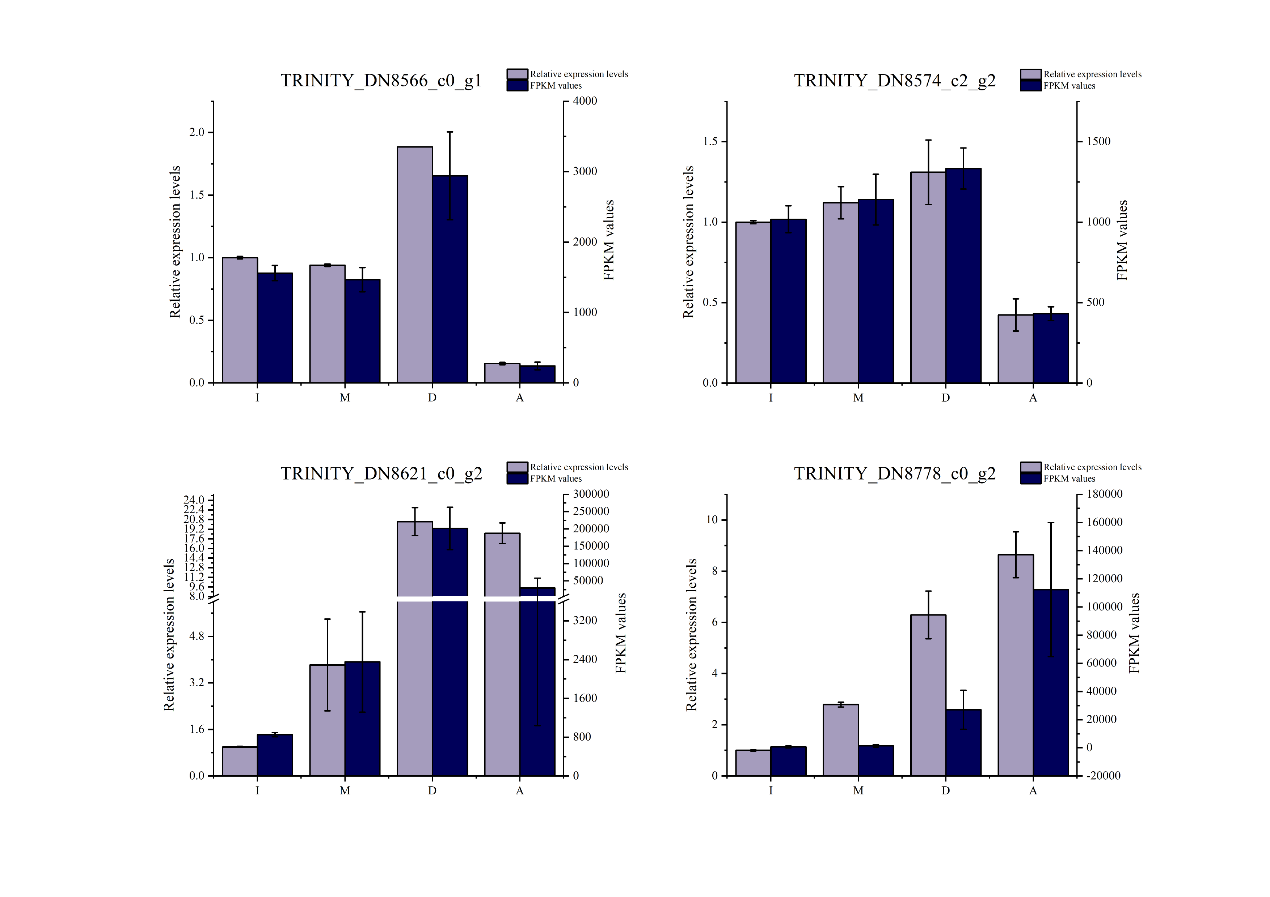


Figure S5. Comparison of qRT-PCR analysis of gene expression with RNA-seq data in stages I, M, D, and A of fruiting body development in *C. comatus.* The Y axis shows the relative mRNA expression levels.
